# Role of *Bacillus* sp. TF-1 in the Degradation and Detoxification of Trifluralin

**DOI:** 10.3390/microorganisms13030520

**Published:** 2025-02-27

**Authors:** Haiyan Ni, Yue Ye, Weiwei He, Qing Chen, Zhong’er Long, Yunhong Huang, Long Zou, Xueqin Fu

**Affiliations:** 1 Nanchang Key Laboratory of Microbial Resources Exploitation & Utilization from Poyang Lake Wetland, College of Life Sciences, Jiangxi Normal University, Nanchang 330022, China; nihaiyan16@163.com (H.N.); 15630863102@163.com (Y.Y.); hwweiwi@163.com (W.H.); longzhonger@163.com (Z.L.); sallyyunhong@163.com (Y.H.); zoulong@jxnu.edu.cn (L.Z.); 2 College of Life Sciences, Zaozhuang University, Zaozhuang 277160, China; chenqing8686@126.com

**Keywords:** trifluralin, microbial degradation, *Bacillus* sp. TF-1, degradation fate, detoxification

## Abstract

Trifluralin, a widely utilized dinitroaniline herbicide, has emerged as a prevalent environmental contaminant that poses significant risks both to ecosystems and to human health. Microbial degradation represents the primary pathway for preventing trifluralin accumulation in the environment. Although much work has been conducted on the microbial breakdown of trifluralin, numerous challenges persist regarding the identification of efficient degrading strains, the elucidation of the metabolic pathways involved, and the application of bioremediation techniques. In this study, *Bacillus* sp. TF-1, a strain isolated from a paddy field that can utilize trifluralin as a source of carbon and energy, was applied. Remarkably, it eliminated 86.7% of 100 mg/L trifluralin within 6 h, and 99.7% of trifluralin was eliminated within 48 h. UPLC–MS analysis suggested that trifluralin degradation occurred first through mono-nitroreduction, followed by further nitroreduction and trifluoromethyl oxidation; trifluralin could also be metabolized through complete nitroreduction and N-dealkylation. Furthermore, *Bacillus* sp. TF-1 effectively mitigated the severe toxicity of trifluralin to sensitive crops. These findings not only expand the repertoire of efficient trifluralin-degrading microorganisms but also increase our understanding of trifluralin biodegradation pathways and highlight the biological importance of employing microbes to eradicate trifluralin residues from the environment.

## 1. Introduction

The use of herbicides is an effective means to control weeds, maximize grain production, and keep crops suitable for human consumption. Dinitroanilines are selective, broad-spectrum, high-efficiency herbicides that are typically used as preemergent treatment agents in a wide variety of agroeconomically important crops and occupy a very important position among chemical herbicides [1]. Trifluralin (α,α,α-trifluoro-2,6-dinitro-N,N*-*dipropyl-*p*-toluidine, chemical formula C_13_H_16_F_3_N_3_O_4_) was the first dinitroaniline herbicide developed and has been on the market for 60 years since its commercialization in 1964. To date, trifluralin remains one of the two most popular dinitroaniline herbicides used, with pendimethalin being the other, and is among the world’s main varieties of herbicides [2]. This herbicide targets microtubules by binding with unpolymerized tubulin heterodimers, resulting in disturbed mitosis and arrested mitotic cells in telophase, thereby inhibiting root and bud growth and ultimately leading to the death of weeds [3,4]. Trifluralin has been extensively applied to control annual grassy weeds and small-seeded dicotyledonous weeds in fields of cotton, beans, and vegetables and in fruit orchards [5]. In recent years, the sale of trifluralin has remained robust, with approximately 4400 tons applied globally each year [2]. Notably, trifluralin is particularly heavily used in certain regions. For example, it is the most frequently used herbicide in sunflower fields in Turkey, is heavily used in the main cotton-producing areas of China, is the second most commonly used herbicide in Australia, and is among the 25 most applied pesticides in agriculture in the United States [6,7,8,9]. However, as a xenobiotic aromatic compound, trifluralin is physicochemically stable, readily combines with soil organic matter, and is recalcitrant to degradation, making it an herbicide with moderate to long-term persistence in the environment [3,6,10,11]. Some studies have reported that trifluralin can be detected in some environments years after its initial application [10,12,13].

Residual trifluralin persists in the atmosphere, soils, water, and sediments, and it represents a considerable hazard for the environment and humans [13,14]. Trifluralin shows a high level of toxicity to aquatic organisms (notably to invertebrates and fish) [15,16,17], poses phytotoxicity to some crops and nontarget microbial species [18,19,20], exhibits cytotoxicity and genotoxicity, and is considered mutagenic, with potential teratogenic and fetal toxicity linked to its commercial product nitrosodipropylamine [21,22,23,24]. Consequently, the United States Environmental Protection Agency (USEPA) (1996) has classified trifluralin into group C: possibly carcinogenic to humans [25]. Trifluralin is among the top five most hazardous pesticides of concern to public health, and its use has been banned in the European Union (EU) in early 2008 [26,27,28,29]. Despite these concerns, trifluralin remains one of the most preferred herbicides globally due to its favorable cost–performance ratio. Therefore, there is a pressing need to specifically expand our knowledge of the environmental fate of trifluralin to accurately evaluate the ecological safety and health implications of its continued use at the global scale.

Generally, the dissipation of trifluralin is governed by the contributions of both biotic and abiotic reactions, primarily volatilization, photodegradation, and biodegradation. Microorganisms mainly drive its degradation and transformation [3]. To date, some types of bacteria and fungi have been isolated that degrade trifluralin [3,30,31,32,33,34]. For example, Erguven et al. [7] studied the trifluralin degradation abilities of 11 identified bacteria and fungi by using chemical oxygen demand (COD) as the index. Among bacteria, the best removal of trifluralin was achieved by *Bacillus simplex*, with 95% removal in 5 d, and that among fungi was achieved by *Metacordyceps chlamydosporia*, with 80% removal in 5 d. Three fungal isolates, *Aspergillus carneus*, *Fusarium oxysporum*, and *Trichoderma viride*, showed good ability to degrade 200 mg/L trifluralin over 10 d, with less than 10% trifluralin recovered from the culture media and *T. viride* showing the highest degradation rate of 97% [35]. Studies have shown that microbial strains degrade trifluralin mainly through nitroreduction, N-dealkylation, and cyclization to benzimidazole, and a few metabolic pathways have been proposed [31,32,35,36,37,38,39]. However, to our knowledge, there is little information regarding the efficient degrading strains and metabolic pathways of this compound by pure microbial isolates, since trifluralin is a poor substrate for naturally occurring microbes because of the presence of two nitro groups and a trifluoromethyl group in the structure, which is not frequently found in natural molecules. Moreover, few studies have focused on the application of microbes to detoxify trifluralin or bioremediate media with trifluralin residues. Pure cultures, especially strains with efficient degradation ability, are vital for monitoring the biodegradation and transformation processes of trifluralin residues, analyzing the environmental fate, and detoxifying and environmentally remediating this contaminant. Hence, screening for microbes that degrade trifluralin efficiently or produce enzymes/enzyme systems that degrade trifluralin may prove environmentally profitable at present, and new discoveries related to novel biodegradation pathways can help us to understand, predict, and monitor the fate of trifluralin in the environment.

In this study, *Bacillus* sp. TF-1, which is highly efficient at degrading trifluralin, was isolated from paddy soil. Three metabolic products were identified, revealing that trifluralin is consumed rapidly by strain TF-1 through the reduction of nitro groups, oxidation of trifluoromethyl groups, and didealkylation of propyl groups. Furthermore, strain TF-1 effectively alleviated the toxic effects of trifluralin on sensitive crop species. These findings supplement our knowledge of efficient trifluralin-degrading strains, broaden our understanding of the microbial degradation steps and environmental fate of trifluralin, and reveal the biological importance of applying microbial resources to eliminate the hazards caused by residual trifluralin.

## 2. Materials and Methods

### 2.1. Chemical Reagents and Media

All the herbicides and nitroaromatic compounds used, including trifluralin (99% purity), pendimethalin (99% purity), butralin (99% purity), oryzalin (99% purity), fluoroglycofen-ethyl (99% purity), mesotrione (99% purity), *S*-metolachlor (99% purity), *sym-diphenylcarbazide* (98% purity), 2-nitrophenol (98% purity), and 4-nitrophenol (99% purity), were purchased from Sigma-Aldrich (Shanghai, China). The methanol and acetonitrile used were of chromatography grade, and all other chemical reagents used were of analytical grade. All the above herbicides and aromatic compounds were first dissolved in methanol and then filtered through a 0.22 μm organic filter membrane before use. The compositions of the mineral salt medium (MSM) and Luria–Bertani (LB) broth are shown in Table S1.

### 2.2. Isolation of Trifluralin-Degrading Bacteria from Paddy Soil

The traditional enrichment culture method was used to isolate trifluralin-degrading bacteria. Approximately 90.0 g of soil sample was collected from the 0–20 cm surface layer of a paddy field in Xinjian District, Nanchang City, Jiangxi Province. Based on the World Reference Base for Soil Resources (WRB), the soil belongs to Hydragric Anthrosols. After collecting, the soil particles were thoroughly blended. Subsequently, 5.0 g of the soil was weighed out and inoculated into 95 mL of MSM supplemented with 50 mg/L trifluralin and cultured in the dark on a rotary shaker at 150 rpm and 30 °C for 5 d. Subsequently, the enrichment cultures were transferred into fresh MSM (10%, *V*/*V*) containing 100, 150, 200, or 250 mg/L trifluralin. The final enrichment culture was plated on LB agar plates supplemented with 100 mg/L trifluralin via the gradient dilution method. Single colonies were selected, purified, and tested for their ability to degrade trifluralin via high-performance liquid chromatography (HPLC) analysis. In the end, an efficient trifluralin-degrading bacterium, designated TF-1, was obtained. The micromorphology of strain TF-1 was observed via Gram staining and transmission electron microscopy (TEM, H-7650, HITACHI, Hitachi, Ibaraki, Japan). The sequence of the 16S rRNA gene of strain TF-1 was obtained via a method described by Ni et al. [40], and the sequence similarity was analyzed via the EzBioCloud Database (https://www.ezbiocloud.net/, accessed on 23 August 2023). The phylogenetic tree of the 16S rRNA gene sequence of strain TF-1 (GenBank accession number PQ538520) and related strain types was constructed via the neighbor-joining method via MEGA software (version V) [41].

### 2.3. Microbial Degradation of Trifluralin by the Isolated Strain TF-1

The isolate was incubated in LB broth at 30 °C and 150 rpm, and the contents were harvested at the late-exponential phase by centrifugation at 5000 rpm for 5 min. The bacterial cells were washed twice with fresh MSM and then resuspended in the same medium, after which the seed cells were obtained. The degradation of trifluralin was performed in 20 mL of MSM containing 100 mg/L trifluralin as a carbon and energy source, and the initial cell density of strain TF-1 at 600 nm (OD_600_) was adjusted to 0.8. Inactivated bacteria inoculated in MSM supplemented with 100 mg/L trifluralin composed the control group. Each treatment was performed in triplicate, and the assessment of degradation was performed at 30 °C and 150 rpm in the dark. The biomass (OD_600_) of strain TF-1 and the concentrations of residual trifluralin in the cultures were evaluated at specific times.

Using the same procedures, the influences of different culture conditions, including temperature (20–42 °C), pH (4.0–10.0), initial cell density (OD_600_ = 0.2–1.0), and initial trifluralin concentration (50–250 mg/L), on trifluralin degradation by strain TF-1 were determined. The effect of each environmental condition on trifluralin degradation was evaluated under optimal degradation conditions with respect to the other environmental variables (temperature, pH, and initial cell density). In addition, the ability of strain TF-1 to degrade other dinitroaniline herbicides (pendimethalin, butralin, and oryzalin) and nitroaromatic compounds (*S*-metolachlor, fluoroglycofen-ethyl, mesotrione, 2-nitrophenol, 4-nitrophenol, and *sym*-dibenzoylhydrazine) was tested. Strain TF-1 with an initial biomass of OD_600_ = 0.8 was inoculated into 20 mL of MSM containing 100 mg/L of each degradation substrate. The control group was set up in the same manner as described above. Each treatment was conducted in triplicate, and degradation assessments were carried out at 30 °C and 150 rpm for a duration of 48 h.

### 2.4. Metabolite Identification and Metabolic Pathway Analysis

The culture medium samples collected at different timepoints were mixed with an equal volume of dichloromethane and then shaken vigorously. The mixtures were allowed to stand until the aqueous and organic phases were completely separated. The organic phase was dried with anhydrous Na_2_SO_4_ and evaporated to dryness. All the treated samples were ultimately redissolved in methanol and filtered through 0.22 µm organic membrane filters. The samples from the degradation of other substrates were treated in the same way. The intermediate products of trifluralin degradation were identified via ultra-performance liquid chromatography coupled with Q-TOF mass spectrometry (UPLC-Q-TOF/MS) analysis.

### 2.5. Detoxification of Trifluralin by Strain TF-1 in Sensitive Crops

The physiological toxicity of trifluralin toward sensitive crops and the detoxifying ability of strain TF-1 were assessed via the germination and growth of pakchoi (*Brassica campestris* L. ssp. *chinensis* Makino), alfalfa (*Medicago sativa* L.), and spinach (*Spinacia oleracea* L.) as index crops. The seeds of pakchoi and alfalfa were cultured with Hoagland solution in Petri dishes. Strain TF-1 was preinoculated, collected, washed, and resuspended in PBS buffer (pH 7.0), and the biomass (OD_600_) was adjusted to 1.0, which was referred to as the seed culture of strain TF-1. One group of crop seeds was treated with trifluralin at different concentrations (25, 50, and 100 mg/L), and another group was treated with the same amounts of trifluralin (25, 50, and 100 mg/L) and a 10% seed culture of strain TF-1. Seeds without trifluralin and bacterial cells were used as the blank controls. Each treatment was carried out in six replicates. All the seeds were cultured at 25 °C, the germination rate of the seeds was calculated, and the growth status of the seedlings was observed after 7 d. In addition, the length and dry weight of each sprout were calculated on the 7th day.

To assess the detoxifying effects of strain TF-1 on the growth of pakchoi and spinach seedlings, the soil used was sieved through a 2 mm sieve, sterilized, and dried. The concentration of trifluralin applied to the soil was 1904 ga.i./hm^2^, the amount of strain TF-1 premixed with dry soils was 10% of the initial OD_600_ = 1.0 solution, and the soils for the control treatments were prepared by applying only PBS buffer (pH 7.0). The crop pots (7 cm × 7 cm) were filled with 60.0 g of the soils from different treatments, and seedlings of each crop with roughly the same growth were planted separately at a depth of 4 cm. Seedling growth was observed under four different planting environments: pot 1, no addition of trifluralin or strain TF-1; pot 2, addition of strain TF-1; pot 3, addition of both trifluralin and strain TF-1; and pot 4, addition of trifluralin. Each treatment was replicated six times. All the pots were maintained at room temperature under natural sunlight throughout the 5-week experimental period and were hand-watered daily, but overwatering and herbicide leaching were avoided during this period.

### 2.6. Analytical Methods

The biomass of strain TF-1 (OD_600_) was measured via a UV spectrophotometer (UV-2600, Shimadzu, Kyoto, Japan), and spectral scanning analysis (200−600 nm) via this UV spectrophotometer was used as a preliminary qualitative analysis method for the degradation of these aromatic compounds. Subsequently, qualitative and quantitative analyses of all the substrates that strain TF-1 could degrade were carried out via HPLC (LC-20 AT, Shimadzu, Japan). Separation was performed using a Shimadzu Shim-pack GIS C18 column (4.6 mm × 250 mm, 5 μm) at 35 °C. The mobile phase consisted of methanol/ultrapure water (85/15, *V*/*V*) with a flow rate of 1 mL/min, and the detection wavelength was set as 240 nm for the dinitroanilines, 230 nm for fluoroglycofen-ethyl, 260 nm for mesotrione, and 210 nm for *sym-diphenylcarbazide*. An Acquity UPLC system (Waters) and a Xevo G2-XS Q-TOF/MS system (Waters, Milford, MA, USA) (UPLC-Q-TOF/MS) were used to identify the metabolites of trifluralin after degradation. Chromatographic separation was performed using a Waters Acquity UPLC BEH C18 column (2.1 mm × 100 mm, 1.8 μm), and the column temperature was maintained at 35 °C. The mobile phase consisted of solvent A (0.1% formic acid in 5 mM ammonium formate) and solvent B (100% acetonitrile). A gradient elution procedure was applied for compound separation at a flow rate of 0.3 mL/min under the following conditions: 0–1 min, 2% B; 1–8 min, 2–40% B; 8–9 min, 40–90% B; 9–12 min, 90% B; 12–12.1 min, 90–2% B; and 12.1–15 min, 2% B. The injection volume was 5 μL, and each sample was detected under positive mode by electrospray ionization (ESI) with a mass scanning range of 100–1200 *m*/*z*. UNIFI 2.0 software was used for data analysis.

## 3. Results

### 3.1. Isolation and Characterization of the Trifluralin-Degrading Strain

**Figure 1:**
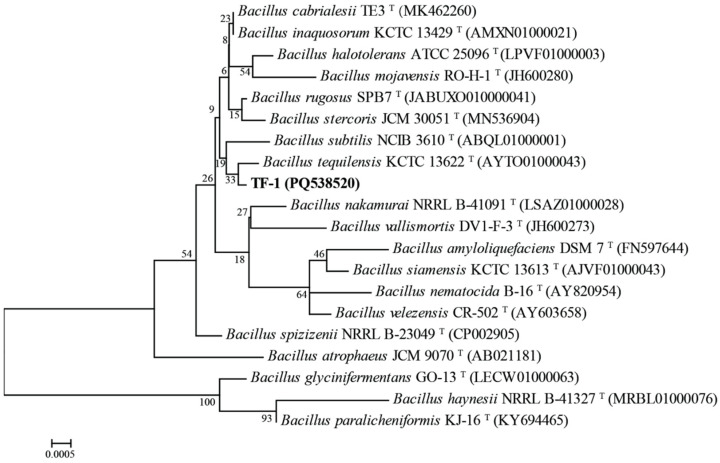
Phylogenetic tree based on the 16S rRNA gene sequences of strain TF-1 and related species.

After the enrichment culture, 11 different colonies were obtained, but only two strains, designated TF-1 and TF-5, were verified to degrade trifluralin, and strain TF-1 exhibited greater degradation ability and was chosen for further study. Strain TF-1 was found to be a rod-shaped, peritrichous, capsule-shaped, Gram-positive, and spore-forming bacterium, with a bacterial size of approximately 0.8–0.9 μm × 2.8–2.9 μm (Figure S1A,B). The morphology of colonies of strain TF-1 on LB plates was circular, off-white, with neat edges, and was sticky, moist, smooth, raised, and easy to pick up during the exponential period, but when the bacterium reached the later stages of growth, the colonies became dry, wrinkled, and convex and were not easily disturbed (Figure S1C,D). Online alignment analysis revealed that the 16S rRNA gene sequence of strain TF-1 displayed 99.86% similarity with the 16S rRNA gene sequences of *Bacillus tequilensis* KCTC 13,622 ^T^, *B. cabrialesii* TE3 ^T^, and *B. inaquosorum* KCTC 13,429 ^T^ and clustered on a subbranch with *B. tequilensis* KCTC 13,622 ^T^ (Figure 1). In summary, strain TF-1 was identified as a member of *Bacillus*.

### 3.2. Biodegradation of Trifluralin by Bacillus sp. TF-1

**Figure 2:**
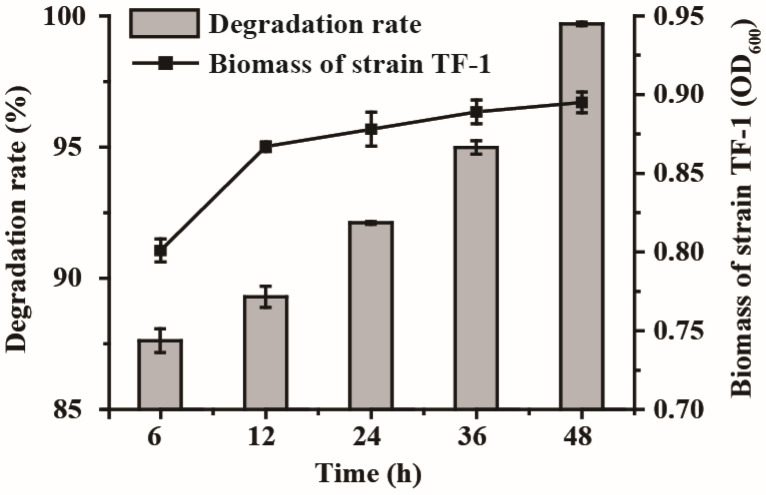
Trifluralin degradation curve and biomass variation in strain TF-1 at specific times in MSM supplemented with 100 mg/L trifluralin.

As degradation progressed, the concentration of trifluralin in the cultures gradually decreased (Figure 2). The initial concentration of 100 mg/L trifluralin was reduced by 87.6% at 6 h, and the concentration of trifluralin in the culture was determined to be 0.3 mg/L at 48 h. Meanwhile, the biomass of strain TF-1 also increased gradually, from the initial OD_600_ = 0.8 to the final OD_600_ = 0.9 (Figure 2). These data suggested that strain TF-1 could utilize trifluralin as a carbon and energy source for growth and showed high efficiency in degrading this herbicide.

### 3.3. Degradation Characteristics of Bacillus sp. TF-1

**Figure 3:**
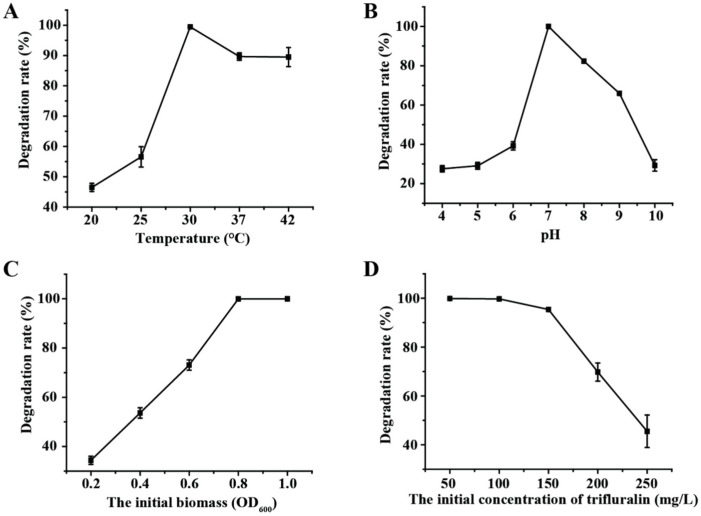
Trifluralin degradation behavior of *Bacillus* sp. TF-1 under different environmental conditions. (**A**) Temperature; (**B**) pH; (**C**) initial biomass (OD_600_); (**D**) initial concentration of trifluralin.

The effects of different environmental factors on trifluralin degradation by strain TF-1 were explored. The optimal temperature for trifluralin degradation by strain TF-1 was 30 °C (Figure 3A). Over 48 h, the degradation rate of trifluralin by strain TF-1 gradually increased from 46.5% to 100% as the temperature increased from 20 °C to 30 °C and decreased from 100% to 89.5% as the temperature increased from 30 °C to 42 °C (Figure 3A). The capacity of strain TF-1 to degrade trifluralin was significantly influenced by the initial pH of the culture (Figure 3B). The degradation rate was more than 66.0% at pH 7.0–9.0; however, it decreased dramatically at pH values of 4.0, 5.0, 6.0, and 10.0, with rates of 27.5%, 29.1%, 39.2%, and 29.2%, respectively. The effect of the initial biomass of strain TF-1 on degradation efficiency is displayed in Figure 3C. As the initial biomass (OD_600_) of strain TF-1 increased from 0.2 to 1.0, the residual concentration of trifluralin in the culture decreased from 65.6 mg/L to 0 within 48 h, and the optimal initial biomass of strain TF-1 was measured to be OD_600_ = 0.8. Trifluralin was completely degraded within 48 h when its initial concentration was less than 100 mg/L; however, when the initial trifluralin concentration was increased to 150 mg/L and 200 mg/L, the removal rates of this herbicide were slightly reduced to 95.4% and 69.8%, respectively (Figure 3D). However, biodegradation was significantly inhibited at high concentrations of trifluralin (≥250 mg/L). This might have occurred due to the inability of strain TF-1 to completely detoxify and degrade trifluralin at high concentrations, so the toxic effects of trifluralin on the cells of strain TF-1 were not mitigated, which led to a reduction in the degradation rate.

The ability of strain TF-1 to degrade three other prominent dinitroanilines and other types of herbicides as well as aromatic compounds containing similar structures was characterized under optimal conditions. The results showed that strain TF-1 could degrade 100 mg/L pendimethalin, butralin, and oryzalin, with the removal rates of the three tested dinitroaniline herbicides being 99.9%, 99.2%, and 99.8%, respectively, over 48 h (Figure S4A,B,C). In addition, strain TF-1 effectively degraded the diphenyl ether herbicide fluoroglycofen-ethyl (Figure S4D), the aromatic compound *sym*-diphenylcarbazide (Figure S4E), and the triketone herbicide mesotrione (Figure S5), but it could not degrade 2-nitrophenol, 4-nitrophenol, or *S*-metolachlor (Figures S6–S8).

### 3.4. Identification of Intermediate Products and Analysis of the Metabolic Pathways in Bacillus sp. TF-1

During the degradation process, three intermediate products were detected via UPLC-Q-TOF-MS (Figure S2). Trifluralin was observed at a retention time (RT) of 10.47 min, with a peak at *m*/*z* 336.1164 ([M+H]^+^), which was associated with a fragment at *m*/*z* 202.0611 (M–C_3_H_9_O_2_) (Figure S2A). Product M1, with an RT of 10.32 min, was detected with a molecular ion *m*/*z* of 306.1408 ([M+H]^+^), and its fragments were observed at *m*/*z* 240.0737 (M–C_2_H_8_FN), *m*/*z* 230.0908 (M–C_3_H_6_FN), *m*/*z* 225.0510 (M–C_3_H_11_FN), and *m*/*z* 215.0694 (M–C_2_H_9_F_3_) (Figure S2B). Thus, M1 was proposed to be a mono-nitro-reduced product of trifluralin, α,α,α-trifluoro-5-nitro-N,N-dipropyl-toluene-3,4-diamine (NAT). The molecular ion of M2 was observed at the peak of *m*/*z* 274.1506 ([M+Na]^+^) with an RT of 7.52 min, which showed four characteristic fragment ions of *m*/*z* 160.0872 (M–C_4_H_11_O_2_), *m*/*z* 146.0713 (M–C_5_H_3_O_2_), *m*/*z* 145.0752 (M–C_4_H_12_NO_2_), and *m*/*z* 133.0639 (M–C_6_H_14_O_2_) (Figure S2C). Similarly, M3, whose RT was 5.52 min, had a molecular ion at *m*/*z* 192.0726 ([M+H]^+^) and a characteristic fragment ion at *m*/*z* 175.0473 (M–NH_2_) (Figure S2D). Therefore, M2 and M3 were identified as 3,5-diamino-*p*-(dipropylamino) benzoic acid (DACT) and α,α,α-trifluoro-toluene-3,4,5-triamine (TAT), respectively.

**Figure 4:**
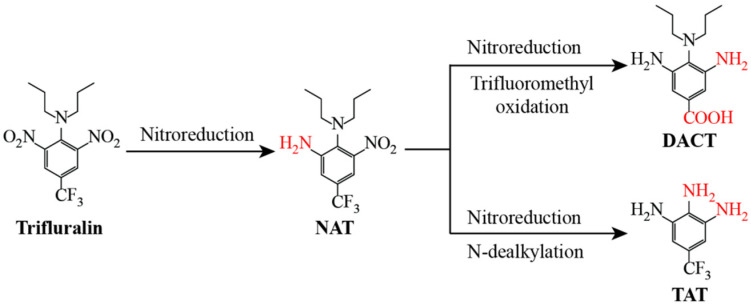
Proposed partial metabolic pathway of trifluralin degradation by strain TF-1. The red groups in the chemical structures indicates the targets of the degradation reactions and the new groups formed by the biotransformation. NAT: α,α,α-trifluoro-5-nitro-N,N-dipropyl-toluene-3,4-diamine; DACT: 3,5-diamino-*p*-(dipropylamino)-benzoic acid; TAT: α,α,α-trifluoro-toluene-3,4,5-triamine.

On the basis of the identified metabolites of trifluralin, it was determined that *Bacillus* sp. TF-1 initially degraded trifluralin by mono-nitroreduction to produce NAT, and then it utilized NAT through two different pathways: in one, NAT was consumed by further nitroreduction and oxidation of the trifluoromethyl group to form DACT; in the other, M1 was nitroreduced and completely N-dealkylated to generate TAT (Figure 4).

### 3.5. Detoxifying Role of Bacillus sp. TF-1 in Trifluralin-Sensitive Crops

**Figure 5:**
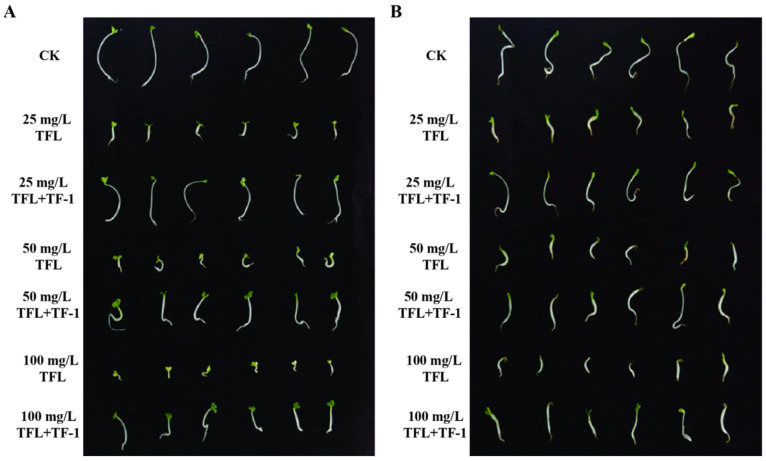
Germination status of seeds of trifluralin-sensitive crops after 7 d of hydroponic growth. (**A**) Pakchoi; (**B**) alfalfa. CK, seeds without any other treatments; 25 mg/L TFL, seeds treated with 25 mg/L trifluralin; 25 mg/L TFL+TF-1, seeds treated with both 25 mg/L trifliralin and the cells of strain TF-1; 50 mg/L TFL, seeds treated with 50 mg/L trifluralin; 50 mg/L TFL+TF-1, seeds treated with both 50 mg/L trifluralin and the cells of strain TF-1; 100 mg/L TFL, seeds treated with 100 mg/L trifluralin; 100 mg/L TFL+TF-1, seeds treated with both 100 mg/L trifluralin and the cells of strain TF-1.

The germination of pakchoi seeds subjected to different treatments is shown in Figure 5A. All treated seeds sprouted. However, compared with those of the control group with neither trifluralin nor strain TF-1, the germination of seeds treated with only trifluralin was significantly inhibited, and the symptoms of toxicity associated with trifluralin residues included stunted growth with short and atrophic sprouts, swollen primary roots, and yellow cotyledons. The higher the concentration of trifluralin was, the stronger the inhibitory effect, and the more obvious the toxicity symptoms. In the presence of strain TF-1, the germination of seeds treated with trifluralin was restored; in particular, the germination of seeds treated with 25 mg/L trifluralin was essentially the same as that of the control group. As shown in Figure 5B, the effect of trifluralin on alfalfa seed germination was similar to that on the seeds of pakchoi, and the growth trend improved with the addition of strain TF-1. The lengths and dry weights of seedlings in all the treatments are presented in Table S2. Trifluralin clearly affected the seeds of pakchoi and alfalfa, with significant reductions in seedling length and dry weight, but strain TF-1 effectively prevented these reductions. The results are consistent with the trends observed in seed germination.

**Figure 6:**
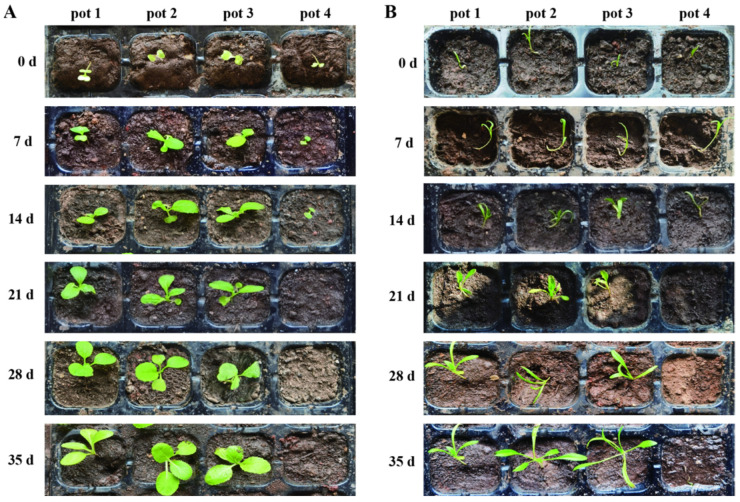
Growth processes of trifluralin-sensitive crops under different treatments. (**A**) Pakchoi; (**B**) spinach.

In addition, the growth status of pakchoi and spinach seedlings in different pots was observed and recorded every 7 d (Figure 6A,B). The results revealed that within 35 d, the growth trend of pakchoi and spinach in pot 2 did not differ substantially from that in pot 1 (the control group), indicating that strain TF-1 had no harmful effect on the growth of the two crops. The growth trend of the crops in pot 3 was also similar to that in pot 1; however, the growth of pakchoi and spinach in pot 4 was significantly inhibited, and the affected crops were characterized by limited growth from 0 to 7 d and the death of all the seedlings at 21 d. On the basis of these results, it was inferred that trifluralin could cause serious phytotoxicity in sensitive crops, such as pakchoi and spinach, whereas strain TF-1 can effectively alleviate the phytotoxic effects of trifluralin on sensitive crops, restoring their growth.

## 4. Discussion

Herbicide use in farming systems has raised concerns not only due to the negative impacts of herbicides on ecological safety and human health but also due to uncertainties regarding the ultimate fate of these compounds in the environment. Trifluralin is a widely used dinitroaniline herbicide, and its persistence makes it a substantial threat to the ecosystem and to human health, which has led to the use of cost-effective treatment technologies for herbicide degradation and/or removal. Microbial degradation plays a major role in controlling the fate of trifluralin in soil and is generally considered desirable from both environmental and agricultural perspectives. Many reports suggest that enrichment culture is a useful and effective method for obtaining microorganisms with degradation functionality [42]. Here, a bacterial strain, TF-1, capable of degrading the xenobiotic compound trifluralin, was isolated from a paddy field via this traditional enrichment culture method and further identified as *Bacillus* sp. Members of *Bacillus* are common Gram-positive bacteria that widely exist in soil, water, plant surfaces, and other natural environments. Owing to their robustness and environmental adaptability, *Bacillus* species are very important microbial resources that have been widely used in medicine, food, the chemical industry, agriculture, environmental protection, and other fields. Many studies have characterized the functional degradation of various recalcitrant compounds via *Bacillus* species. For example, *B. subtilis* Y3 has been reported to degrade pendimethalin, trifluralin, butralin, and oryzalin [38,39]; *B. amyloliquefaciens* SL-7 has been shown to degrade lignin efficiently [43]; *Bacillus* sp. FE-1 has been found to degrade fomesafen both in liquid media and in soil [44]; and *B. subtilis* A1 has been characterized as a degrader of crude oil [45]. Further mining of resources of this genus will help us understand its biological diversity and strong biodegradation potential.

To date, some bacteria and fungi have been identified as trifluralin degraders. The capacities of five identified bacteria, six identified fungi, and their mixtures for trifluralin biodegradation were determined with COD as the measured parameter in agitated culture media [7]. Among the bacterial mixture (91%), fungal mixture (84%), and the bacterial + fungal mixture, the latter resulted in the highest degradation rate of 93% toward 311 mg/L trifluralin over 5 d. For pure isolates, the highest and lowest removal rates of trifluralin in bacteria were achieved by *B. simplex* B1 (95%) and *Clostridium tetani* B5 (86%) after 5 d, respectively, and in fungi, the highest degradation rate was exhibited by *M. chlamydosporia* F4 (80%); the lowest degradation rate was shown by *Penicillium simplicissimum* F2, which was only 59%. The effect of *Pseudomonas fluorescens* on the biodegradation of trifluralin in four media types was studied, and the highest biodegradation rate (64%) was obtained in media supplemented with carbon + nitrogen at 72 h after inoculation [34]. In addition, Zayed et al. [35] demonstrated that three fungi, *A. carneus*, *F. oxysporum*, and *T. viride*, could degrade more than 90% of the initial 200 mg/L trifluralin within 10 d, and *T. viride* achieved the best removal rate of 97%. In this study, *Bacillus* sp. TF-1 was found to remove 87.6% of 100 mg/L trifluralin within 6 h and 95.4% of 150 mg/L trifluralin within 48 h. These data indicate that strain TF-1 has higher degradation efficiency toward trifluralin than other reported trifluralin-degrading strains.

Despite the efforts of many researchers on the microbial degradation of trifluralin, many questions remain to be addressed, such as the influence of environmental factors on degradation rates and the metabolism and environmental fate of trifluralin [3]. Therefore, the degradation behavior of *Bacillus* sp. TF-1 toward trifluralin was analyzed to achieve better remediation of trifluralin-polluted sites via bioremediation deliberately in future. Strain TF-1 maintained effective degradation ability under different environmental conditions. This strain removed more than 46% of 100 mg/L trifluralin at 20–42 °C and retained more than 66% of the same degradation rate with 100 mg/L trifluralin when the pH was between 7.0–9.0. The trifluralin degradation rate was more than 95% when the initial concentration of trifluralin was no more than 150 mg/L. In addition, strain TF-1 showed a broad degradable substrate spectrum and effectively degraded pendimethalin, butralin, oryzalin, fluoroglycofen-ethyl, mesotrione, and *sym-diphenylcarbazide,* which provides important information for the application of *Bacillus* in the bioremediation of herbicide-contaminated environments.

Trifluralin, pendimethalin, butralin, and oryzalin are the four predominant dinitroaniline herbicides, and they have similar chemical structures, physicochemical properties, and broad-spectrum herbicidal activities. The previously reported pendimethalin-degrading strain *B. subtilis* Y3 also degraded the same four dinitroaniline herbicides; it showed higher catalytic activity toward pendimethalin and butralin but lower catalytic activity toward trifluralin and oryzalin [38,39]. However, the identified butralin-degrading strain *Sphingopyxis* sp. HMH could degrade butralin and pendimethalin but not trifluralin or oryzalin [46]. The different degradation efficiencies/abilities of these strains toward the four dinitroaniline herbicides may be partially due to the differences between the N-terminal substituents and the C-4 substituents on the aromatic ring of the four dinitroanilines. As shown in Figure S3, pendimethalin and butralin have only one N-alkyl group, whereas trifluralin and oryzalin possess two N-alkyl groups; the C-4 substituents on the aromatic rings of pendimethalin and butralin are methyl and *tert*-butyl, respectively, whereas the C-4 substituents on the aromatic rings of trifluralin and oryzalin are trifluoromethyl and aminosulfonyl groups, respectively. It is possible that the presence of two N-alkyl groups, a trifluoromethyl group (trifluralin) and an aminosulfonyl group (oryzalin), results in greater steric hindrance during enzyme–substrate interactions. Notably, the degradation rates of strain TF-1 toward 100 mg/L trifluralin, pendimethalin, butralin, and oryzalin were essentially the same, all of which were greater than 99%, indicating that the different substituents had no obvious influence on the degradation activity of strain TF-1 and that there may be a novel enzyme/enzyme system responsible for the catalysis of the four dinitroaniline herbicides. This result is significantly different from that obtained with other characterized dinitroaniline-degrading strains. Taken together, these findings indicate that strain TF-1 has greater application potential in the bioremediation of media polluted with residual herbicides.

**Figure 7:**
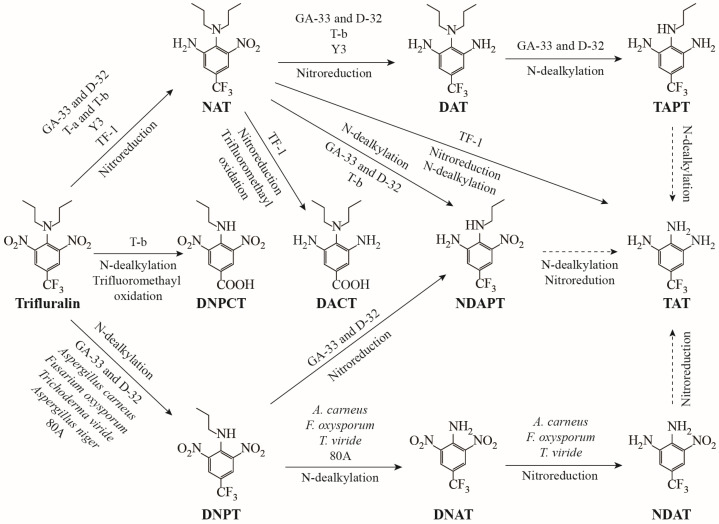
Nitroreduction and N-dealkylation degradation pathways of trifluralin by pure isolates. NAT: α,α,α-trifluoro-5-nitro-N,N-dipropyl-toluene-3,4-diamine; DAT: α,α,α-trifluoro-N,N*-*dipropyl-toluene-3,4,5-triamine; TAPT: α,α,α-trifluoro-N*-*propyl-toluene-3,4,5-triamine; DACT: 3,5-diamino-*p*-(dipropylamino)-benzoic acid; DNPCT: 3,5-dinitro-4-propylamine-benzoic acid; DNPT: α,α,α-trifluoro-2,6-dinitro-N-propyl-*p*-toluidine; DNAT: α,α,α-trifluoro-2,6-dinitro-*p*-toluidine; NDAT: α,α,α-trifluoro-5-nitro-toluene-3,4-diamine; NDAPT: α,α,α-trifluoro-5-nitro-N*-*propyl-toluene-3,4-diamine; TAT: α,α,α-trifluoro-toluene-3,4,5-triamine. The dotted arrows indicate that these processes have not been reported in the literature. Strains GA-33 and D-32 were derived from reference [31]; strains T-a and T-b were derived from reference [32]; *Aspergillus carneus*, *Fusarium oxysporum* and *Trichoderma viride* were derived from reference [35]; *A. niger* was derived from reference [36]; strain 80A was derived from reference [37]; strain Y3 was devised from references [38,39].

Previous studies have shown that nitroreduction and N-dealkylation are two major and key microbial degradation processes for trifluralin under aerobic conditions (Figure 7). Both *Bacteroides ruminicola* subsp. *brevis* GA-33 and *Lachnospira multiparus* D-32 initially degraded trifluralin through mono-nitroreduction and N-terminal mono-dealkylation, and further nitroreduced or N-dealkylated their corresponding intermediate metabolites to generate α,α,α-trifluoro-N*-*propyl-toluene-3,4,5-triamine (TAPT) and α,α,α-trifluoro-5-nitro-N*-*propyl-toluene-3,4-diamine (NDAPT) [31]. In *Alcaligenes* sp. T-a, only the mono-nitroreduction product of trifluralin, NAT, was detected [32]. However, in the presence of *Moraxella* sp. T-b, trifluralin first underwent mono-nitroreduction to form NAT; subsequently, NAT was transformed into α,α,α-trifluoro-N,N*-*dipropyl-toluene-3,4,5-triamine (DAT) by further nitroreduction or to NDAPT by N-terminal mono-dealkylation [32]. Interestingly, 3,5-dinitro-4-propylamine-benzoic acid (DNPCT), the mono-dealkylation and trifluoromethyl oxidation product of trifluralin, was also detected in the degradation extract of strain T-b. Four fungi, *Candida* sp. 80A, *A. carneus*, *F. oxysporum*, and *T. viride*, completely N-dealkylated trifluralin to produce α,α,α-trifluoro-2,6-dinitro-*p*-toluidine (DNAT), and the latter three fungi further nitroreduced one nitro group of DNAT to form α,α,α-trifluoro-5-nitro-toluene-3,4-diamine (NDAT) [35,37]. In conclusion, the microbial degradation process of trifluralin has not been elucidated past the first 1–3 steps until now, and the corresponding final degradation products obtained from nitroreduction, N-dealkylation, and trifluoromethyl oxidation are TAPT, NDAPT, NDAT, and DNPCT, respectively. However, the subsequent transformation products have not been reported. The mono-nitroreduced product, NAT, the dinitroreduced and trifluoromethyl oxidized product, DACT, and the completely nitroreduced and N-dealkylated product, TAT, were detected during the degradation of trifluralin by strain TF-1. *Bacillus* sp. TF-1 first degraded trifluralin by mono-nitroreduction to form NAT, which then underwent further nitroreduction and trifluoromethyl oxidation, converting NAT to DACT. Trifluralin is a xenobiotic compound, with a chemical structure containing dinitroaromatic hydrocarbon and a trifluoromethyl group, and the latter group is never observed in nature [47]. No abiotic degradation processes or photoreactions have led to the removal of the trifluoromethyl group [3]. To our knowledge, among microbes, only two strains can exploit the trifluoromethyl group of trifluralin, the earlier strain T-b and the present *Bacillus* sp. TF-1. However, the transformed product DACT in *Bacillus* sp. TF-1 is different from DNPCT produced by strain T-b, which was obtained by complete nitroreduction and trifluoromethyl oxidation. In addition, transformation of NAT to TAT via strain TF-1 also occurred after nitroreduction and two rounds of N-dealkylation. Notably, TAT was produced by dinitroreduction and N-terminal didealkylation, which is the transformation product of TAPT, NDAPT, or NDAT, and was detected among the microbial degradation products of trifluralin for the first time (Figure 7). Taken together, the newly identified DACT and TAT supplement our knowledge of the metabolites of trifluralin produced by pure isolates and enhance our understanding of the environmental fate of trifluralin.

Bioremediation methodologies, in which microorganisms are used to eliminate organic environmental contaminants into forms that have less or no toxicity, have attracted considerable attention and have indicated to be efficient tools for decontaminating pesticide-polluted sites [48]. Although considerable research has been conducted on the degradation of trifluralin in soil or by pure cultures, there are few reports on trifluralin bioremediation by microorganisms. In the present study, the mitigation of the toxic effects of trifluralin in sensitive crops by *Bacillus* sp. TF-1 was evaluated. Exposure to trifluralin led to a significant slowdown in the germination process of the tested sensitive crop seeds. Concurrently, there were marked decreases in both the length of the seedlings and their dry weight. Similar toxicity symptoms were previously documented by Chowdhury et al. [19] and Chen et al. [49]. In addition, trifluralin could cause severely inhibited growth in the tested seedlings, which could ultimately culminate in their death. However, strain TF-1 effectively ameliorated the significant toxic effects of trifluralin on these sensitive crops, suggesting that microbial degradation is an effective way to remediate trifluralin-contaminated media and that strain TF-1 is promising for the treatment of trifluralin pollution. However, there are still some limitations on the effectiveness of microbial remediation processes in practice, which are related to the type and concentration of pollutants, their interactions with indigenous microorganisms, and environmental conditions. Therefore, more efforts should be made to maximize the utilization of microbial resources to remediate contaminated sites.

## 5. Conclusions

Here, strain TF-1, identified as *Bacillus* sp., was isolated from paddy soil via traditional enrichment culture. This bacterium can utilize trifluralin as a carbon and energy source for growth and degrade 87.6% of 100 mg/L trifluralin within 6 h and 95.4% of 150 mg/L trifluralin within 48 h. Three degradation products were identified, suggesting that trifluralin was degraded by nitroreduction, trifluoromethyl oxidation, and N-dealkylation. Among the three identified products, DACT and TAT, which were newly detected metabolites during trifluralin microbial degradation, help us to understand more details of the biodegradation processes of trifluralin, thus deepening the understanding of the environmental fate of trifluralin. Moreover, strain TF-1 effectively relieved the strong toxicity of trifluralin to sensitive crops. These findings offer new insights for the sustainable biodegradation of trifluralin.

## Data Availability

The original contributions presented in the study are included in the article/Supplementary Material, further inquiries can be directed to the corresponding author.

## Supplementary Materials

The following supporting information can be downloaded at https://www.mdpi.com/article/10.3390/microorganisms13030520/s1: Table S1: Media used in this study; Table S2: Physiological indexes of trifluralin-sensitive crops after germination; Figure S1: The morphologies of strain TF-1, (A) the morphology observed by TEM, (B) the morphology observed by Gram staining, (C) the colonies on the LB broth at the exponential period of strain TF-1, (D) the colonies on the LB broth at the later growth stage of strain TF-1; Figure S2: UPLC–MS analysis of the degradation products of trifluralin produced by stain TF-1, (A) trifluralin, (B) M1, α,α,α-trifluoro-5-nitro-N,N-dipropyl-toluene-3,4-diamine (NAT), mono-nitroreduction of trifluralin, (C) M2, 3,5-diamino-*p*-(dipropylamino) benzoic acid (DACT), dinitroreduction and trifluoromethyl oxidation product of trifluralin, (D) M3, α,α,α-trifluoro-toluene-3,4,5-triamine (TAT), dinitroreduction and N-terminal didealkylation product of trifluralin; Figure S3: Chemical structures of the major dinitroaniline herbicides trifluralin, pendimethalin, butralin, and oryzalin; Figure S4: HPLC analysis of degradation of pendimethalin, butralin, oryzalin, fluoroglycofen-ethyl, and *sym*-dibenzoylhydrazine by strain TF-1, (A) pendimethalin; (B) butralin; (C) oryzalin; (D) fluoroglycofen-ethyl; (E) *sym*-dibenzoylhydrazine; Figure S5: HPLC analysis of meotrione degradation by strain TF-1; Figure S6: Spectral scanning analysis of *S*-metolachlor by strain TF-1; Figure S7: Spectral scanning analysis of 2-nitrophenol by strain TF-1; Figure S8: Spectral scanning analysis of 4-nitrophenol by strain TF-1.
