# Supplementary material for: Role of Bacillus sp. TF-1 in the Degradation and Detoxification of Trifluralin
Source: Microorganisms. 2025 Feb 27;13(3):520. doi: 10.3390/microorganisms13030520 (PMC11945047; doi:10.3390/microorganisms13030520)
Supplement: Supplementary file 1 [file microorganisms-13-00520-s001.zip › Supplementary materials 2025-02-17.pdf]

Supplementary Materials

Role of *Bacillus* sp. TF-1 in the degradation and detoxification of trifluralin

Haiyan Ni [a], Yue Ye [a], Weiwei He [a], Qing Chen [b], Zhonger Long [a], Yunhong Huang [a], Long Zou [a], Xueqin Fu [a,*]

[a] *Nanchang Key Laboratory of Microbial Resources Exploitation & Utilization from Poyang Lake Wetland, College of Life Sciences, Jiangxi Normal University, Nanchang 330022, Jiangxi Province China*

[b] *College of Life Sciences, Zaozhuang University, Zaozhuang 277160, Shandong Province China*

* Corresponding author

E-mail address: 002730@jxnu.edu.cn (Xueqin Fu)

Tel: +86-0791-88120396; Fax: +86-0791-88120396

Supplementary tables

Table S1. Media used in this study

Medium	Competent *
mineral salt medium (MSM)	NH_4Cl (1.0 g/L), NaCl (0.5 g/L), K_2HPO_4 (1.5 g/L), KH_2PO_4 (0.5 g/L), and $MgSO_4 \cdot 7H_2O$ (0.2 g/L), supplemented with trifluralin as the sole carbon source.
Luria−Bertani (LB) broth	tryptone (10.0 g/L), yeast extract (5.0 g/L), and NaCl (10.0 g/L)

*The agar was added at 15.0 g/L for solid medium. Both media were sterilized at 121 °C for 30 min before use.

Table S2. Physiological indexes of trifluralin-sensitive crops after germination

	Stem length (cm)		Dry weight (mg)	
	pakchoi	alfalfa	pakchoi	alfalfa
CK	4.96±0.32	3.94±0.36	0. 62±0.01	0. 28±0.02
25 mg/L TFL	2.92±0.40	1.52±0.11	0. 43±0.01	0. 22±0.01
25 mg/L TFL+TF-1	4.52±0.37	2.56±0.18	0. 65±0.04	0. 31±0.03
50 mg/L TFL	1.54±0.39	1.52±0.23	0.23±0.05	0. 12±0.01
50 mg/L TFL+TF-1	3.9±0.24	2.02.±0.22	0.41±0.05	0. 24±0.05
100 mg/L TFL	1.13±0.10	1.38±0.18	0.24±0.07	0. 08±0.02
100 mg/L TFL+TF-1	2.32±0.26	1.94±0.23	0.56±0.05	0. 26±0.04

Note: CK, crop seeds with no treatments of trifluralin and strain TF-1; 25 mg/L TFL, crop seeds treated with 25 mg/L trifluralin; 25 mg/L TFL+TF-1, crop seeds treated with both 25 mg/L trifluralin and strain TF-1; 50 mg/L TFL, crop seeds treated with 50 mg/L trifluralin; 50 mg/L TFL+TF-1, crop seeds treated with both 50 mg/L trifluralin and strain TF-1; 100 mg/L TFL, crop seeds treated with 100 mg/L trifluralin; 100 mg/L TFL+TF-1, crop seeds treated with both 100 mg/L trifluralin and strain TF-1.

Supplementary figures

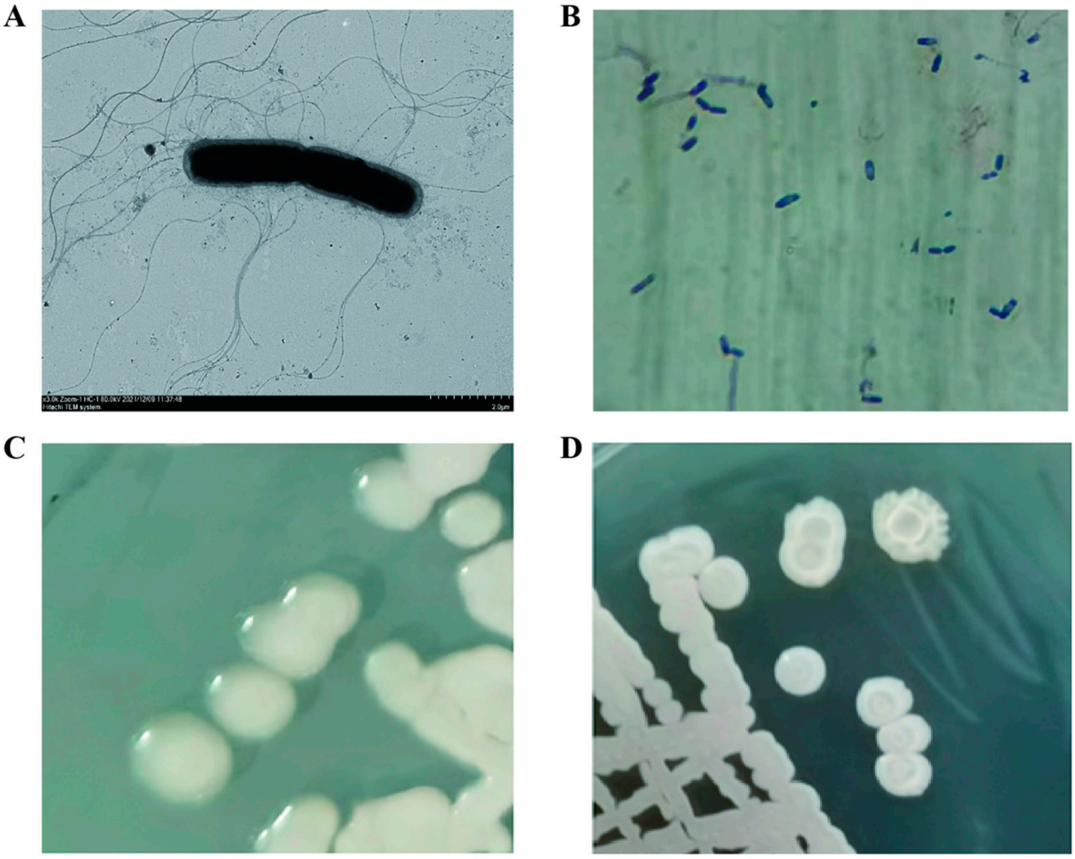

Figure S1. The morphologies of strain TF-1

A, the morphology observed by TEM; B, the morphology observed by Gram staining; C, the colonies on the LB broth at the exponential period of strain TF-1; D, the colonies on the LB broth at the later growth stage of strain TF-1.

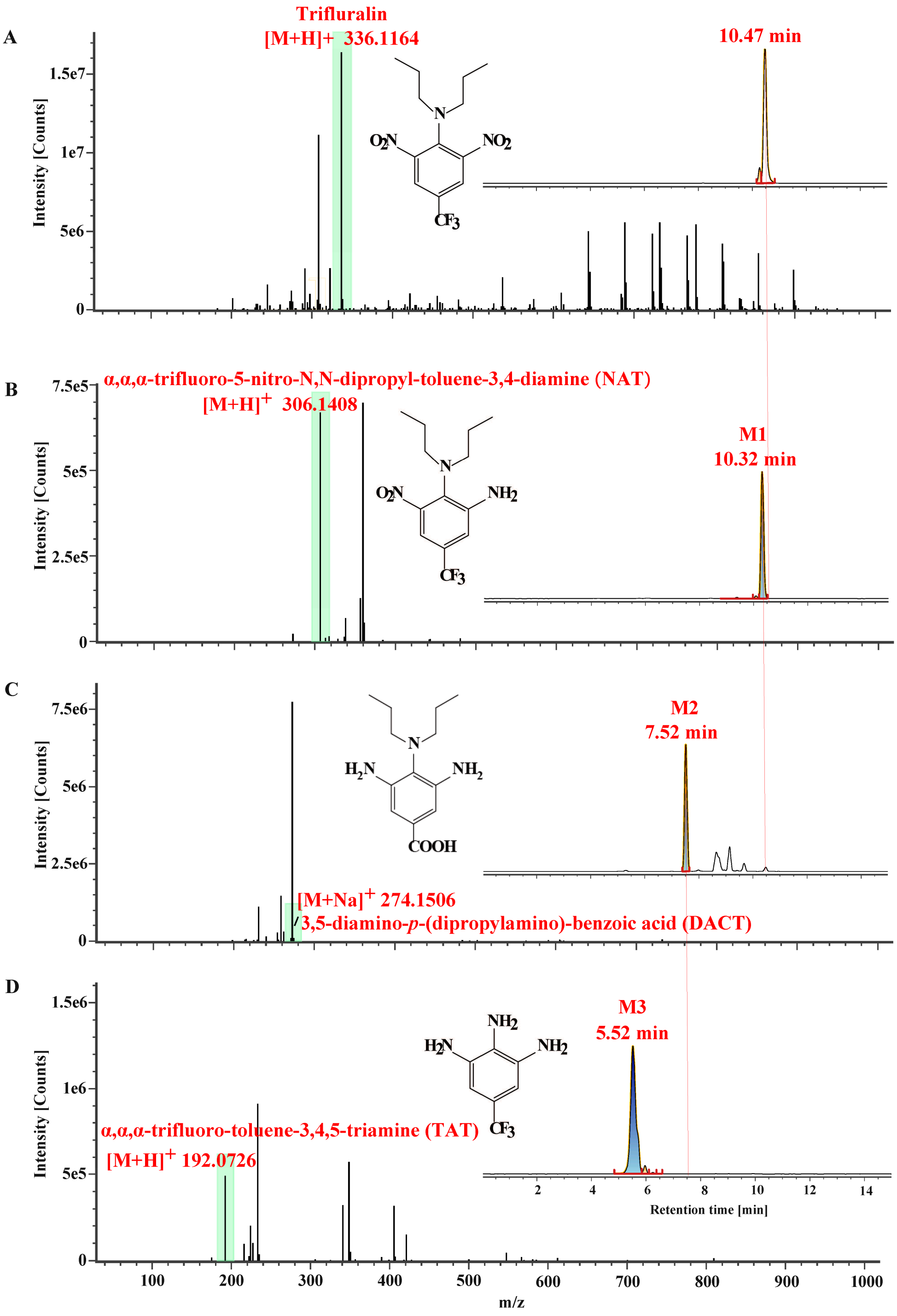

Figure S2. UPLC-MS analysis of the degradation products of trifluralin produced by stain TF-1

A, trifluralin; B, M1, α,α,α-trifluoro-5-nitro-N,N-dipropyl-toluene-3,4-diamine (NAT), mono-nitroreduction of trifluralin; C, M2, 3,5-diamino-*p*-(dipropylamino) benzoic acid (DACT), dinitroreduction and trifluoromethyl oxidation product of trifluralin; D, M3, α,α,α-trifluoro-toluene-3,4,5-triamine (TAT), dinitroreduction and N-terminal didealkylation product of trifluralin.

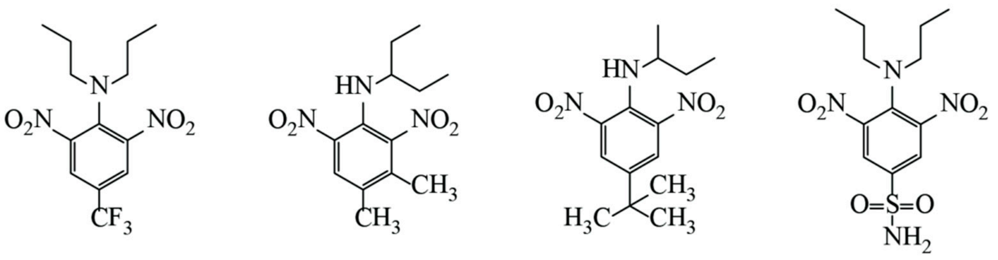

Trifluralin **Pendimethalin** **Butralin** **Oryzallin**

Figure S3. Chemical structures of the major dinitroaniline herbicides trifluralin, pendimethalin, butralin, and oryzalin

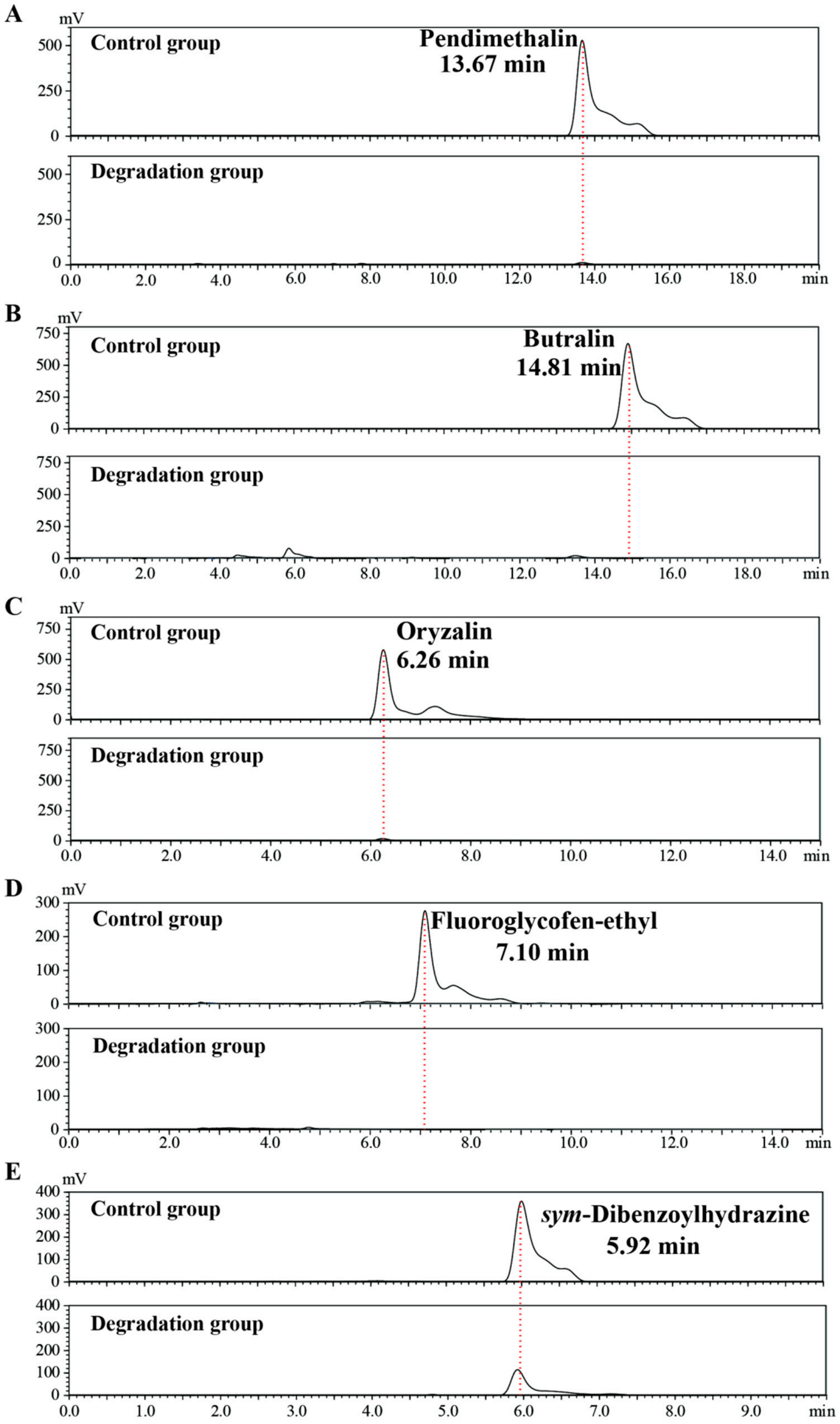

Figure S4. HPLC analysis of degradation of pendimethalin, butralin, oryzalin, fluoroglycofen-ethyl, and *sym*-dibenzoylhydrazine by strain TF-1.

A, pendimethalin; B, butralin; C, oryzalin; D, fluoroglycofen-ethyl; E, *sym*-dibenzoylhydrazine.

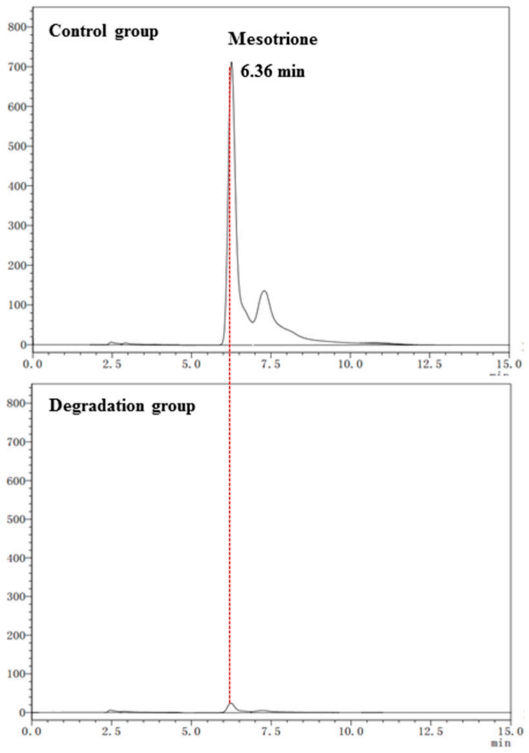

Figure S5. HPLC analysis of meotrione degradation by strain TF-1.

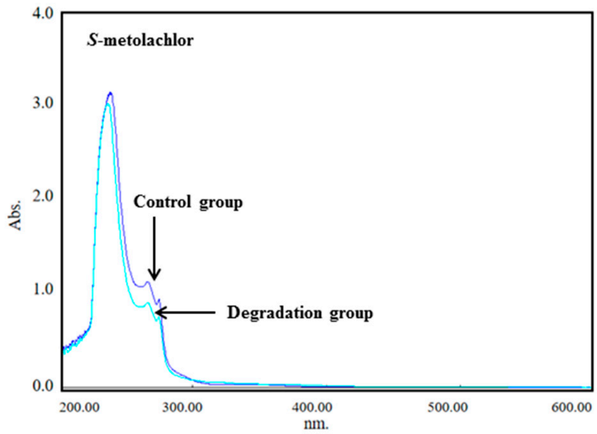

Figure S6. Spectral scanning analysis of *S*-metolachlor by strain TF-1.

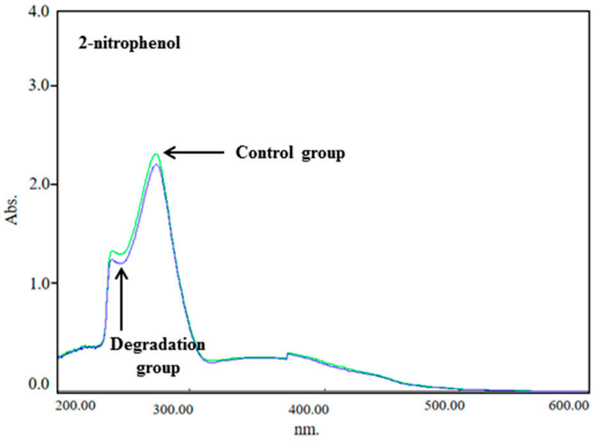

Figure S7. Spectral scanning analysis of 2-nitrophenol by strain TF-1.

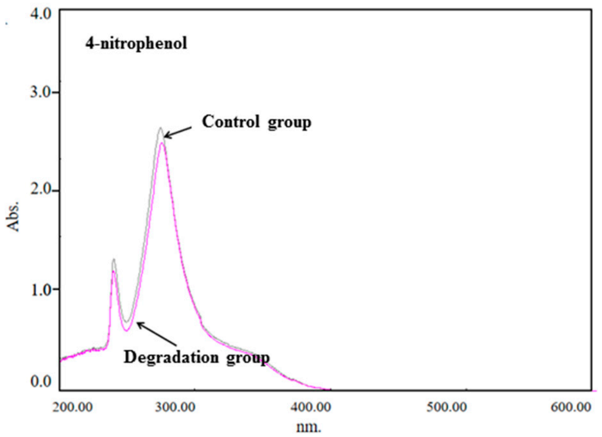

Figure S8. Spectral scanning analysis of 4-nitrophenol by strain TF-1.
