# Supplementary figures and images for: Role of Bacillus sp. TF-1 in the Degradation and Detoxification of Trifluralin
Source: Microorganisms. 2025 Feb 27;13(3):520. doi: 10.3390/microorganisms13030520 (PMC11945047; doi:10.3390/microorganisms13030520)

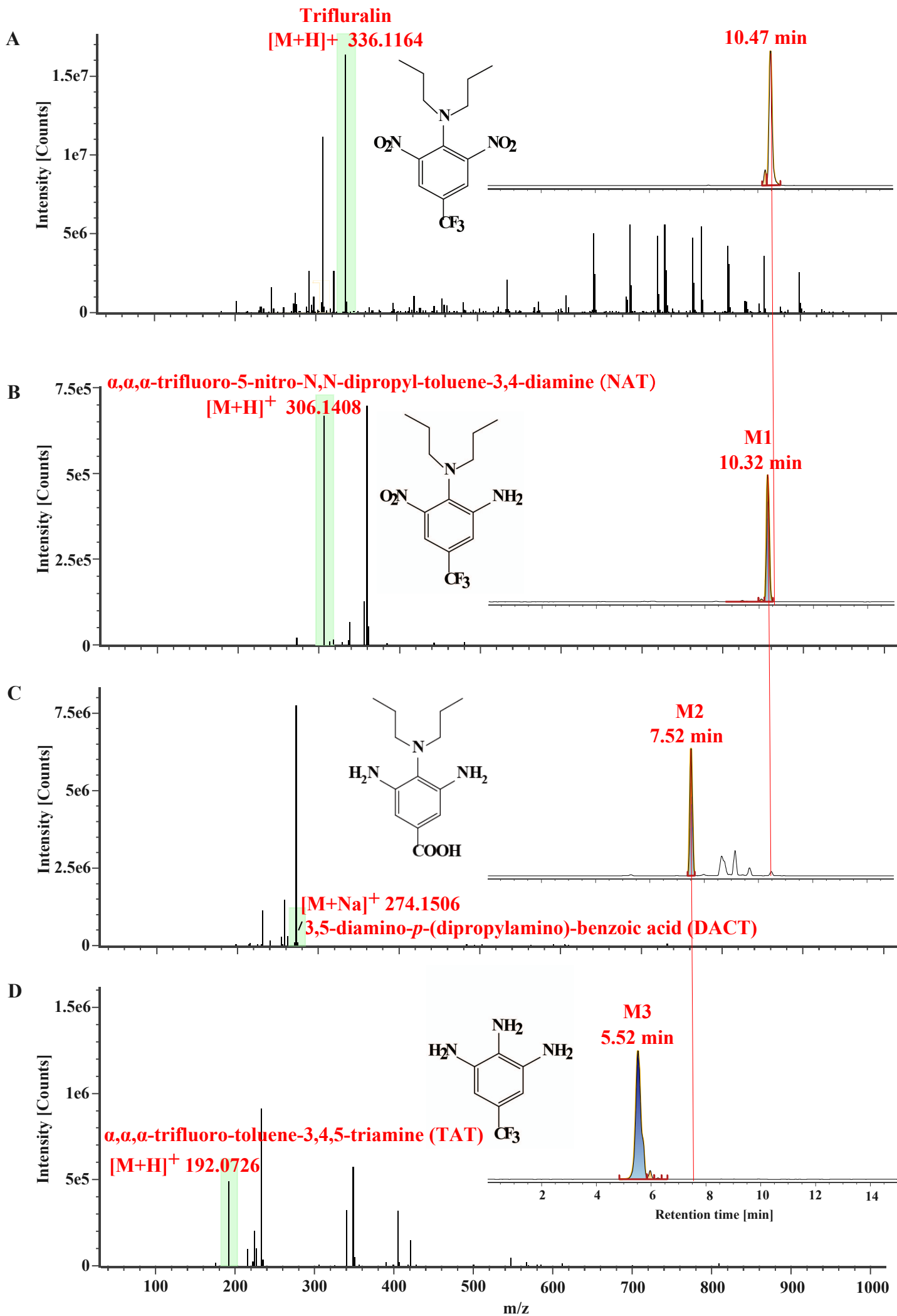

Supplement: Supplementary file 1 [file microorganisms-13-00520-s001.zip › Figure S2.pdf]

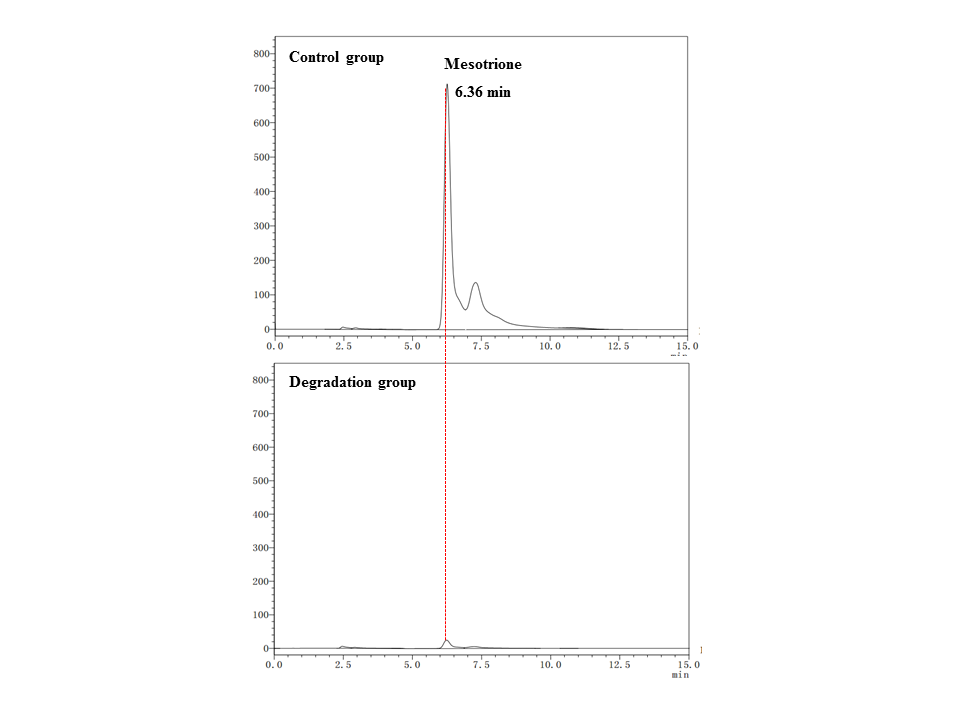

Supplement: Supplementary file 1 [file microorganisms-13-00520-s001.zip › Figure S5.tif]

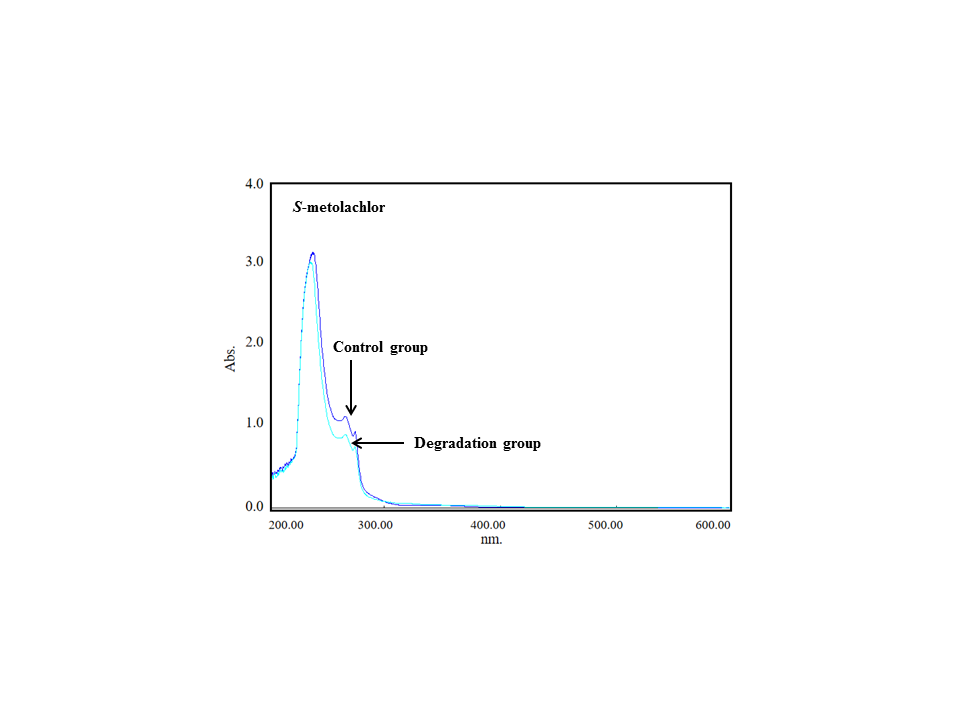

Supplement: Supplementary file 1 [file microorganisms-13-00520-s001.zip › Figure S6.tif]

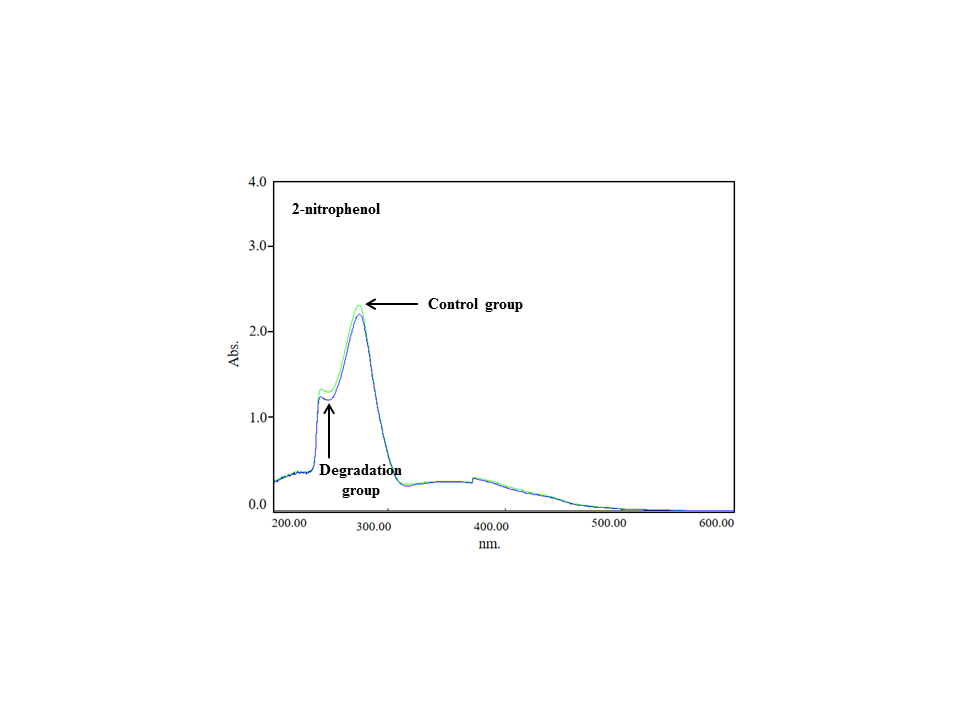

Supplement: Supplementary file 1 [file microorganisms-13-00520-s001.zip › Figure S7.tif]

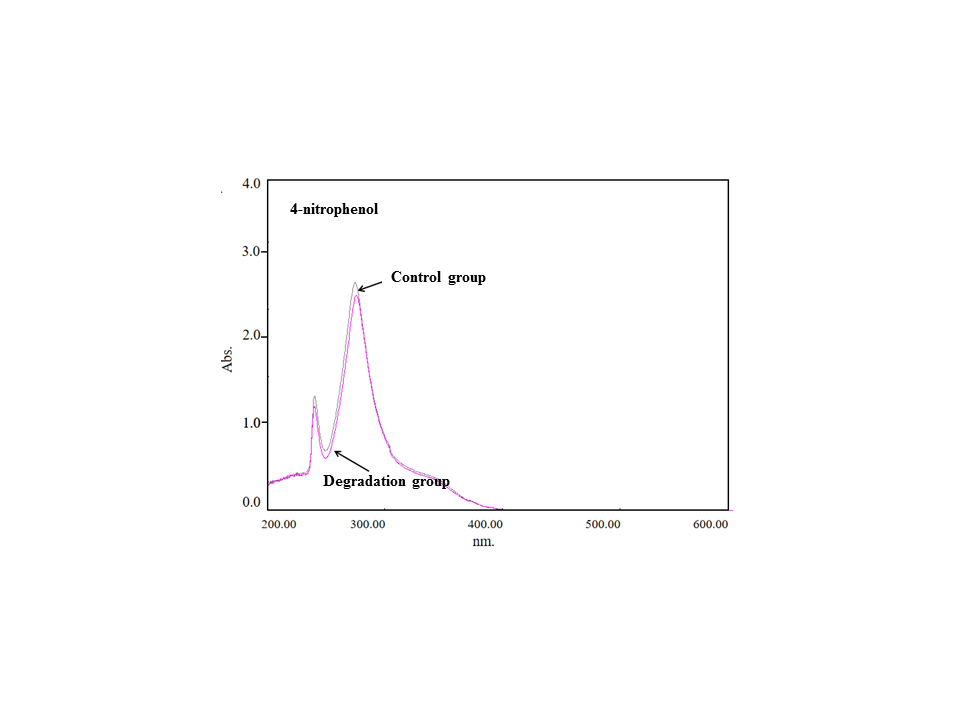

Supplement: Supplementary file 1 [file microorganisms-13-00520-s001.zip › Figure S8.tif]
